# Effects of White Wine Consumption on Weight in Rats: Do Polyphenols Matter?

**DOI:** 10.1155/2017/8315803

**Published:** 2017-10-31

**Authors:** Ana Marija Milat, Ivana Mudnić, Ivica Grković, Nikola Ključević, Mia Grga, Iva Jerčić, Diana Jurić, Danica Ivanković, Benjamin Benzon, Mladen Boban

**Affiliations:** ^1^Department of Pharmacology, University of Split School of Medicine, Split, Croatia; ^2^Department of Anatomy, University of Split School of Medicine, Split, Croatia

## Abstract

**Introduction:**

Effects of white wine and the role of wine polyphenols on weight gain in rats of different age were examined in the 4-week-voluntary-consumption trial.

**Methods and Materials:**

Biochemically characterized standard (low polyphenols, W) and macerated (high polyphenolic content, PW) white wines were compared. One- and three-month-old Sprague-Dawley male rats (*n* = 78) were used. Each age group was subdivided into water-only-drinking controls (C), W, and PW-drinking animals. Daily wine and total liquid consumption, food intake, and body weight were measured, and energy intake and feed efficiency index were calculated.

**Results:**

In both age categories, wine-drinking animals consumed less food and gained less weight in comparison to C (181 ± 2, 179 ± 6, and 201 ± 5 in younger animals and 32 ± 5, 28 ± 6, and 47 ± 4 grams in older animals, resp.), regardless of wine type. Total energy intake was the lowest in PW-drinking animals.

**Conclusion:**

Wine-drinking animals gained less weight in comparison to C, regardless of the wines' polyphenol content. Although our results are indicative of the major role of nonphenolic constituents of the wines (probably ethanol), the modifying role of wine phenolics on weight gain cannot be excluded as the group consuming PW had lower total energy intake than other groups.

## 1. Introduction

Despite large epidemiological evidence, it is still controversial whether alcohol intake represents a risk factor for weight gain and obesity.

Based on recent reviews of epidemiological studies regarding the effect of alcohol consumption on body weight, it seems that only heavy drinking is positively related with weight gain [1, 2]. Also, it appears that the type of alcoholic beverage is an important element in modifying the effect of alcohol consumption on weight gain with wine being regarded as an alcoholic beverage with more favourable effects [3]. Several animal studies, conducted under controlled experimental conditions, found that red wine intake did not cause an increase in body weight, compared to the water-only-drinking rats [4, 5]. Advantageous effects of wine were largely attributed to different biological actions of wine polyphenols. Among these, reduced appetite and/or nutrient absorption, promotion of energy expenditure, or prevention of energy storage have been proposed as potential mechanisms by which wine phenolics may contribute to the antiobesity effects of wine [6].

Because of much higher concentrations of wine polyphenols being found in red than in white wine, practically all studies examining effects of wine consumption on body weight have been conducted using red wine. In order to examine effects of white wine and the role of wine polyphenols on the weight gain in rats, we compared effects of white wines with low and high phenolic content in the 4-week-consumption trial.

Since it was observed that some differences in the impact of alcohol on body weight may be related to age [7], we studied two groups of animals, younger rats in the phase of fast growth and development and older animals that were close to reaching their body weight plateau.

## 2. Materials and Methods

### 2.1. Animals and Diets

**Figure 1:**
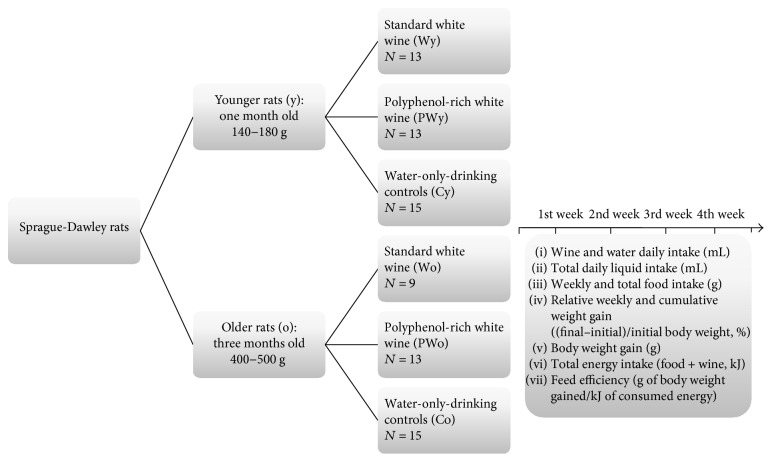
Methodological diagram of the sample, interventions, and main outcomes. W, standard white wine; PW, polyphenol-rich white wine; C, water-only-drinking control animals; y, younger animals; o, older animals.

With respect to the initial body mass and age, two groups of the Sprague-Dawley male rats were used: the younger group (fast-growing animals), one-month-old rats weighing 140–180 g, and the older group (slow-growing animals), approximately 3-month-old rats weighing 400–500 g. Both age groups were further divided into wine-drinking animals and water-only-drinking controls. The wine-drinking animals were offered a standard white wine or polyphenol-rich white wine ad libitum, 24 hours/day for 4 weeks with daily inclusion of tap water for 6 hours. Controls, water-only-drinking animals, were offered tap water without restrictions. Each age group of animals was finally divided into 3 subgroups: water-only-drinking controls (C), standard white wine (W), and polyphenol-rich white wine (PW)-drinking animals (Figure 1). The exclusion criterion for the wine-drinking animals was daily wine intake of less than 7 mL. The fresh beverages were supplied daily in small pet containers with a leak proof nozzle (Ferplast Small Pet Sippy Water Bottle, Castelgomberto, Italy). Fluid intake was recorded daily. The animals were fed a standard pellet diet (Mucedola S.R.L., Settimo Milanese, Milan, Italy) with caloric value of 16.54 kJ/g ad libitum. Body weight and food intake were measured weekly using Grundig KW 4060 Digital Scale (weight range 5 kg). The animals were kept in individual cages under standard temperature and light conditions (22–25°C, 12 h light/dark cycle). All procedures and experimental protocols were in accordance to European Convention on Animal Protection and Guidelines on Research Animal Use and were approved by the Ethics Committee of the School of Medicine University of Split and by the Ethics Committee of the Ministry of Agriculture of the Republic of Croatia.

### 2.2. Wines

The standard white wine used was Graševina, 2015, Krauthaker winery, Croatia. Polyphenol-rich white wine was obtained from the same grape variety within the same vineyard and harvest year by using the traditional Georgian wine production principles. It means that grape juice was first allowed spontaneous fermentation in contact with the hard parts of the grape. Following fermentation, without removing grape seeds and skins, the tanks were air-tight sealed at a constant temperature for 120 days. It resulted in production of white wine with orange or amber hue that was high in phenolic content, similar to red wines [8].

### 2.3. Biochemical Analysis of the Wines

The total phenolics and their flavonoid and nonflavonoid subgroup contents were measured spectrophotometrically. The total phenolic content of the samples was determined by the Folin-Ciocalteu method, and the results were expressed as milligrams of gallic acid equivalents (GAE) per liter. Nonflavonoid compounds were determined by the same method after flavonoid precipitation with formaldehyde, and the flavonoid content was calculated as the difference between total phenolic and nonflavonoid content. Absorbencies were monitored by UV-Vis spectrophotometer (Specord 200, Analytik Jena Inc., Jena, Germany), equipped with a six-cell holder and a thermostatically controlled bath. A more detailed description of the above-mentioned methods has been previously published [9].

Individual polyphenols were identified and quantified by an Agilent HPLC system (type RRLC; Agilent Technologies, Santa Clara, CA) using a ZORBAX SB-C18 analytical column (15 × 2.1 mm, 1.8 *μ*m). Phenolic compounds were identified by their retention times. Quantification was carried out by comparison with external standard calibration curves. Each sample was injected twice into the chromatographic system. A detailed description of the method has been published previously [10]. All analytical-grade chemicals and reagents were obtained from Sigma Aldrich (St. Louis, MO).

### 2.4. Statistical Analysis

The sample size was determined using the program G^∗^Power 3.1 (G^∗^Power, Dusseldorf, Germany). Results were expressed as means ± SEM. Data were compared by one-way ANOVA and post hoc Student *t*-test, using SPSS (version 24) software. The level of significance was set at *P* < 0.05.

## 3. Results

Results of the biochemical analysis of the wines including their caloric value, ethanol and phenolic content, and concentrations of selected individual phenolic compounds are presented in Tables 1 and 2.

Initial body weight among animal groups in both age categories was comparable at the beginning of the treatment. On average, younger animals in three experimental groups weighted 164 ± 4 g (standard white wine, W), 155 ± 3 g (polyphenol-rich white wine, PW), and 147 ± 3 g (water-only-drinking animals, C), whereas weights in corresponding groups in older animals were 453 ± 10 g, 439 ± 9 g, and 417 ± 6 g, respectively.

**Figure 2:**
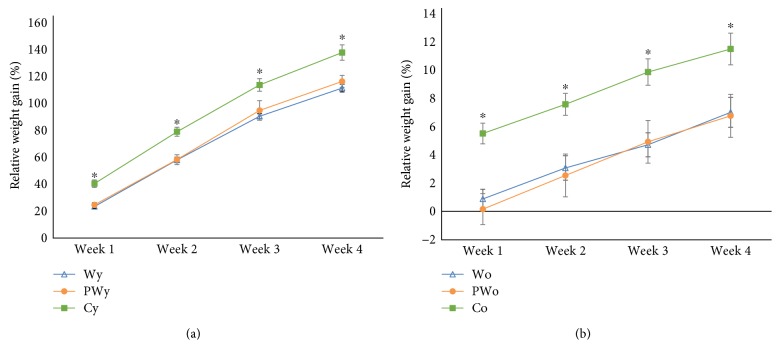
Relative body weight gain in younger (a) and older (b) rats drinking standard (W) or polyphenol-rich (PW) white wine and water-only-drinking controls (C) during 4 weeks. Relative weight gain is expressed as a percentage of initial weight. Final weight gain after 4 weeks is approximately 10 times higher in younger (y) animals than in older (o) ones, so ordinate scales are not matching. ^∗^*P* < 0.05 for water-only-drinking group (C) versus wine-drinking groups (W and PW).

**Figure 3:**
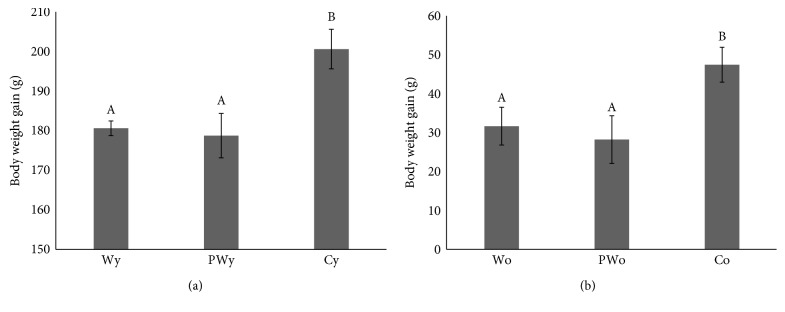
Body weight gain in younger (A) and older (B) rats drinking standard (W) or polyphenol-rich (PW) white wine and water only (C), after 4 weeks. Body weight gain is approximately 4 times higher in younger (y) animals in comparison to older (o) ones, so ordinate scales are not the same. Different letters indicate significant difference between groups (*P* < 0.05).

Expectedly, the rate and the extent of the weight gain in the younger animals were higher than those in the older animals. However, in both age categories, wine-drinking animals generally gained less weight both at weekly and cumulative basis in comparison to control, water-only-drinking animals (Figure 2). In the younger animals, mean body weight gain after 4 weeks of treatment was 181 ± 2 g, 179 ± 6 g, and 201 ± 5 g in W, PW, and C, respectively. In contrast to the water-only-drinking controls, there was no difference in weight gain between the wine-drinking animals (*P* = 0.024 for younger W versus C, *P* = 0.020 for younger PW versus C, and *P* = 0.999 for younger W versus PW, Figure 3(a)). In the older animals, at the same time point, total weight gain was 32 ± 5 g, 28 ± 6 g, and 47 ± 4 g for groups W, PW, and C, respectively. Again, there was no difference in weight gain between the animals drinking either wine (*P* = 0.037 for older W versus C, *P* = 0.029 for older PW versus C, and 0.989 for older W versus PW, Figure 3(b)).

**Figure 4:**
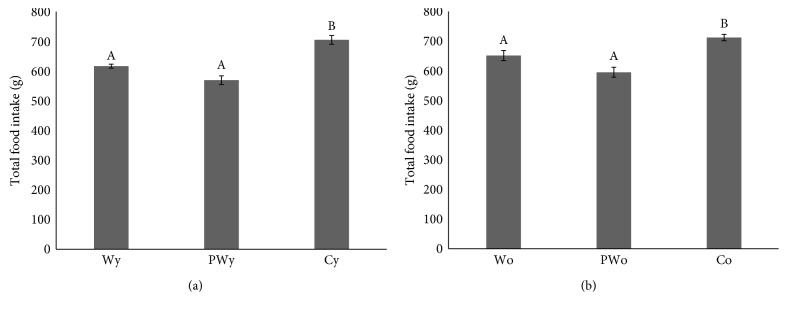
Total food intake in younger (a) and older (b) rats drinking standard (W) or polyphenol-rich (PW) white wine and water only (C), after 4 weeks. Different letters indicate significant difference between groups (*P* < 0.05).

All wine-drinking animals consumed less food at weekly and cumulative basis in comparison to controls, as follows: 154 ± 2 (W), 143 ± 4 (PW), and 176 ± 4 (C) g/week for younger animals and 163 ± 4 (W), 149 ± 4 (PW), and 178 ± 10 (C) g/week for older animals. Data on total food intake after 4 weeks of consumption trial are shown in Figure 4 for both younger and older animals. Following the pattern of weekly food intake, the wine-drinking animals did not significantly differ in their total food intake after 4 weeks regardless of the wine type (*P* = 0.004 for younger W versus C, *P* < 0.001 for younger PW versus C, and *P* = 0.071 for younger W versus PW; *P* = 0.026 for older W versus C, *P* < 0.001 for older PW versus C, and *P* = 0.079 for older W versus PW).

As to daily wine and total liquid intake, W, PW, and C groups ingested similar amounts regardless of the animals' age. Total energy intake (TEI) was calculated as the sum of energetic density of both food and wine, if applicable, in all animal groups after 4 weeks of follow-up. Total energy intake was somewhat lower in younger and older PW groups relative to all other experimental groups.

To assess the amount of body weight gained per kilojoule of consumed energy, we calculated feed efficiency (FE) as the ratio of these two parameters. In general, younger animals had higher FE than older animals as they gained more grams of body weight per kilojoule of consumed energy. However, feed efficiency did not differ between the animals of the same age regardless of the consumed beverage type. Summarized data on daily wine and total liquid intake, total energy intake, and feed efficiency are presented in Table 3.

## 4. Discussion

One of the key findings of our study is a significant association between white wine consumption and lower body weight gain in rats relative to water-only-drinking animals. This is largely in accordance with several other studies that reported similar results in rats and mice following ethanol and red wine consumption [4, 5, 11, 12]. Furthermore, for the first time, we showed that this holds true in both fast-growing younger animals and mature animals which were close to their body weight plateau. As a general rule, wine consumption was associated with decreased food intake in both age categories implying that additional calories provided by wine partially compensate for calories from other foodstuff (Figure 4).

Since no difference was observed between effects of standard and polyphenol-rich white wine on body weight gain and food intake, it appears that wine phenolics are of secondary importance relative to the other constituents of wine, ethanol being the most likely candidate. Namely, this would be in line with Monteiro et al.'s study showing that both red wine- and ethanol-consuming rats gained less weight and ingested less food in comparison to controls, while there was no distinction between the effects of red wine, known to be rich in polyphenols, and ethanol [5].

Interestingly, the total energy intake, provided by calories from food and wine, was lower in polyphenol-rich-wine-drinking animals. This could be in part due to their tendency to consume less food in comparison to standard-wine-drinking animals. Indeed, polyphenols may be involved in appetite control over the central nervous system that monitors the food urge and feeling of satiety [6]. However, there was no difference in feed efficiency among animals of the same age category, regardless of the consumed beverage type. This is simply because animals which ate less gained less weight, so at the end of the experiment they gained similar weight per calorie of energy.

It is important to note that all animals consumed similar total amounts of liquid per day making our subgroups of animals substantially comparable. The seemingly paradoxical fact that younger animals, although weighing three times less than older animals, consume similar amounts of both liquid and food (Table 3 and Figure 4) is just due to their several times higher growth rate. In other words, proportionally higher consumption of food and liquid is necessary to support correspondingly higher metabolic turnover in younger, fast-growing animals. This is also indicated in their (several times) higher feed efficiency ratio (weight gain per calorie, shown in Table 3), relative to older animals. On the other side, steady liquid and food intake in older animals is required for maintaining their basic metabolic needs.

By eliminating differences between test wines, which could be due to differences in the grape variety, growing conditions, terrain, or harvesting time, we were able to determine the influence of wine-making technique and polyphenol content more objectively.

The results of the present study, however, did not determine the essential role of wine phenolics on the animals' weight gain. Although we did not examine the effects of ethanol directly in the present study, our results are indicative of the major role of ethanol, as reported by several different authors [5, 11, 12] who showed that ethanol in general (studied alone or in comparison to red wine) was not associated with body weight gain in the animals.

Generally, moderate drinkers (human or animals) often have significantly lower weight gain in comparison to nondrinkers. Although this may be due to confounding factors (e.g., physical activity), it may also reflect a true physiological effect of alcohol [13].

The energy derived from alcohol differs from other energy sources, since alcohol cannot be stored and is always first to be metabolized. It also has a very high potential to affect metabolic pathways of other nutrients. Intake of alcohol can displace fat and carbohydrates from oxidative metabolism in the liver to a maximum level of 50% the resting value [14]. Indeed, energy stored this way can be more effectively utilized than calories derived from excessive carbohydrate intake [15].

Moreover, thermogenic response of alcohol is rather high and in healthy moderate alcohol consumers is between 15 and 25% of the energy content of the alcohol intake. As compared to fat (~13%) and carbohydrates (~8%), the alcohol energy can be considered as a less usable form of energy [16, 17]. Furthermore, alcohol stimulates sympathetic nervous system and decreases blood glucose by inhibition of liver gluconeogenesis [18]. Finally, when alcohol is consumed in conjunction with a meal, it lowers secretion of the protein leptin more than nonalcohol meals with the same energetic density [19].

Although not focused on underlying mechanisms of alcohol effects on body weight, our study provides additional experimental evidence on this matter. The model of consumption we used is comparable to the model of moderate voluntary wine consumption by Arola et al. where animals had free access to wine, without stressful treatments and non-physiologic consumption of wine (e.g., like in gastric gavage method) [20]. It is important to note that wine consumption by the animals observed in our study in terms of energy intake (approximately 8%) roughly corresponds to the contribution of alcoholic beverages to the total food stuff energy in the human light to moderate alcohol consumers [21].

Finally, the rationale of choosing wine as the alcoholic beverage lies in the fact that it represents an unavoidable constituent of Mediterranean diet. In contrast to red wine, to our best knowledge, the effects of white wine on weight gain in rats have not been studied.

Further studies are still needed to explore the relationship between wine consumption and body weight and to specify the impact of wine phenolics and ethanol. We hope that this simple yet straightforward study may be inspiring to deepen insight into this complex matter.

## 5. Conclusion

The rats drinking white wines gained less weight in comparison to water-only-drinking controls following 4-week-voluntary-consumption trial, regardless of the wine's phenolic content. Although our results are indicative of the major role of the nonphenolic constituents of white wine, the present study cannot exclude the modifying role of wine phenolics on energy metabolism and weight gain as the animals consuming polyphenol-rich white wine had lower total energy intake in comparison to all other experimental groups.

## Figures and Tables

**Table 1:** Caloric value, ethanol, and phenolic content of the tested white wines.

	Ethanol content	Caloric value	Total phenolics	Flavonoids	Nonflavonoids
	vol % (g/L)	kJ/mL	mg GAE/L	mg GAE/L	mg GAE/L
Standard wine	13.0 (102.3)	3.04	305 ± 3^∗^	3^∗^	302 ± 2
Polyphenol-rich wine	13.3 (105)	3.12	2850 ± 35	2477	373 ± 3

Caloric value of tested wines is calculated as the product of ethanol concentration and caloric value of alcohol (29.7 kJ/g). Data on phenolic content are averages of at least three independent measurements and are shown as mean ± SD. Concentration of flavonoids is calculated as the difference between mean concentration of total phenolics and nonflavonoids and is expressed without SD; GAE: gallic acid equivalents; ^∗^*P* < 0.05, Student's *t*-test.

**Table 2:** Concentrations of selected phenolic compounds in tested white wines.

	Gallic acid	(+)-catechin	(−)-epicatechin	Procyanidin B1	Total resveratrol
	mg/L	mg/L	mg/L	mg/L	mg/L
Standard wine	1.45 ± 0.07	1.55 ± 0.07	1.25 ± 0.21	0.85 ± 0.07	0.30
Polyphenol-rich wine	34.75 ± 0.49	109.60 ± 0.28	67.65 ± 1.34	77.55 ± 0.49	2.30

Data are averages of two independent samples and are shown as mean ± SD values.

**Table 3:** Average liquid intake, energy consumption, and feed efficiency among the animal groups.

	Daily wine intake	Total daily liquid intake (wine + water)	TE, total energy intake after 4 weeks	Feed efficiency (BWG/TE)
	mL/d	mL/d	kJ	g/kJ
Wy	10 ± 1	30 ± 1	11088.4 ± 126.4	0.0163 ± 0.0003
PWy	11 ± 1	30 ± 1	10380.1 ± 205.0^∗^	0.0172 ± 0.0004
Cy	n.a.	33 ± 1	11657.5 ± 244.8	0.0173 ± 0.0004

Wo	15 ± 2	29 ± 2	12097.6 ± 315.5	0.0026 ± 0.0004
PWo	11 ± 1	30 ± 1	10808.1 ± 282.4^∗^	0.0025 ± 0.0005
Co	n.a.	32 ± 1	11776.7 ± 179.5	0.0040 ± 0.0004

n.a.: not applicable; BWG: body weight gained; C: control; W: standard white wine; PW: polyphenol-rich white wine; y: younger animals; o: older animals. Results are expressed as means ± SEM. ^∗^*P* < 0.05 for PWy and PWo versus groups within the same age category.
